# Metabolic acid-base disturbances in patients in the emergency department

**DOI:** 10.1186/cc10756

**Published:** 2012-03-20

**Authors:** EM Antonogiannaki, E Lilitsis, D Georgopoulos

**Affiliations:** 1 University Hospital of Heraklion, Greece

## Introduction

The aim of the present study is to determine in unselected patients that visit the emergency department whether the physicochemical approach improves the ability to diagnose acid-base disorders compared with the two commonly used diagnostic approaches; one relying on the plasma bicarbonate concentration (HCO_3_^-^) and anion gap (AG), and the other on the base excess (BE).

## Methods

A prospective observational study took place in the emergency department at a university hospital during the period of March to September 2011. Three hundred and sixty-five patients were included. Arterial and venous samples were drawn for blood gases and a serum biochemical panel, respectively. The decision to collect arterial samples was made by the attending physician in the emergency department who was not involved in the study.

## Results

All patients were admitted to the hospital, while 103 of them (28%) were transferred directly to the ICU. Hypoalbuminemia (serum albumin ≤35 g/l) was observed in 191 patients (52%). The BE and HCO_3_^- ^were normal in 35% and 38% of the total patients, respectively. The corresponding values in patients admitted to the ICU were 41% and 28%. In a significant proportion of patients in whom BE and/or HCO_3_^- ^were normal the physicochemical approach detected the presence of acidifying and/or alkalinizing disturbances. Hypoalbuminemia (metabolic alkalosis) was identified in 45% of patients with normal HCO_3_^- ^and 48% of patients with normal BE. Strong ion difference (SID) acidosis (SID ≤36 mEq/l) was observed in 49% and 44% of patients with normal HCO_3_^- ^and BE, respectively. A high unmeasured strong ion concentration ([XA^-^] ≥8 mEq/l, metabolic acidosis) was observed in 48% of patients with normal HCO_3_^- ^and in 52% of patients with normal BE. Patients in whom hidden acidosis of high unmeasured strong anion type was observed were identified by the common diagnostic approach only using the AG adjusted for hypoalbuminemia (AG_adj _≥13 mEq/l). Patients who were admitted to clinical wards with acidosis, other than hyperchloremic, remained significantly more days in the hospital than those without the disturbance.

## Conclusion

Hypoalbuminemia is a common finding in patients in the emergency department and complicates the interpretation of acid-base data using the common diagnostic approaches. A physicochemical approach may better identify metabolic disturbances in this population.

